# Trustworthiness Over Time on Twitter: Three Critical Periods for the Norwegian Health Authorities and Political Leadership During the COVID-19 Pandemic

**DOI:** 10.1177/20563051231179689

**Published:** 2023-06-08

**Authors:** Jannicke Thinn Fiskvik, Andrea Vik Bjarkø, Øyvind Ihlen

**Affiliations:** 1 SINTEF Digital, Norway; 2 University of Oslo, Norway

**Keywords:** public health authorities, Twitter, trustworthiness, COVID-19, lockdown

## Abstract

Public health authorities and political leaders need to come across as trustworthy in their handling of a crisis like the COVID-19 pandemic. There is, however, little knowledge about how the affordances and dynamics of social media influence perceptions of trustworthiness, especially during a protracted crisis. In this article, we study how Twitter users were discussing the trustworthiness of the Norwegian health authorities and political leadership throughout three periods of partial lockdown during the COVID-19 pandemic. Across all the periods, there was a substantial number of positive comments, but these were outweighed by negative ones. Ability was clearly the most discussed factor for trustworthiness, and many users offered up their lay expertise. Discussions of integrity and benevolence were less frequent and mostly negative when they occurred. An increase in negative comments during the last period might be read as an expression of fatigue, and there was a noted dissatisfaction with the ability of the political leadership. Taken together, the study suggests Twitter to be an arena where users are exposed to arguments and counterarguments in negotiations over ability in particular. Such discussions can intensify as a crisis drags on and are important to grasp for health authorities and political leadership alike. Thus, the study sheds light on the contribution that a socio-technical platform like Twitter makes to the discursive formation of trustworthiness over time, which in turn might function to strengthen or erode public trust in public authorities and political leadership.

## Introduction

Much attention has been devoted to the role of social media during the COVID-19 pandemic. Issues include for instance how social media has spurred vaccine hesitancy and conspiracy theories (Chadwick et al., 2021; Erokhin et al., 2022; Jiang et al., 2021) and influenced trust in scientific expertise (Mihelj et al., 2022; van Dijck & Alinejad, 2020). In this study, we focus on how the *trustworthiness* of the public health authorities and political leadership is discussed. More specifically, we focus on the role of Twitter in this regard, since its “socio-technical infrastructure” (Vicari, 2017, p. 1) has become increasingly important for science communication and political debate. The platform provides citizens with the possibility to engage and express discontent or support with less editorial interference than in traditional news media. Hence, studying Twitter content also gives ready access to the process of the discursive formation of the trustworthiness of health authorities and political leadership that can feed into trust.

In organizational research, trustworthiness is typically defined as the perceptions a trustor has of the ability, integrity, and benevolence of a trustee—a person or an institution (Mayer et al., 1995). Much research has shown how trust is correlated with these facets of trustworthiness (e.g., Colquitt et al., 2007). Still, calls have been issued to unpack the *situational dynamics* of trustworthiness (Baer & Colquitt, 2018). In some situations, certain aspects can be valued over others. For instance, studies have found that integrity was most valued in so-called high-reliability contexts like air traffic control or fire departments. In contrast, in so-called task-related contexts, like assembly line work, benevolence was most appreciated (Colquitt et al., 2011). Furthermore, the ability or competence of experts and political leaders might be judged by observing and commenting on the results of a certain policy and the expert advice that formed the basis of the policy. Research relating to the COVID-19 pandemic has argued that trustworthiness is anchored in the “professional training and proven experience” of the experts and the “transparent methods as well as [. . .] rigorous probing and communal judgment of evidence” of their institutions (van Dijck & Alinejad, 2020, p. 2). The importance of such “perceived ability” in a pandemic situation still needs more research, particularly as it is manifested in social media discussions.

Importantly, situations are not only different but can also *change over time.* Perceptions of trustworthiness are not static entities but are negotiated over time in arenas such as social media. Many studies have investigated how concern and support for different countermeasures have waned over time (e.g., Su et al., 2022), and also how negativity seems to thrive in social media (Ceron, 2015; Humprecht et al., 2020). Thus, the temporal dimension of social media discussions must be grasped (Vicari, 2017) in order to “develop distinct communication strategies at the various stages of a public debate” (van Dijck & Alinejad, 2020, p. 8).

The article focuses on the case of Norway, a country that was spared the worst consequences of the COVID-19 pandemic (see World Health Organization [WHO], 2022). Yet periods of strict measures still had major societal consequences in terms of mental health, social life, and the economy (NOU 2022:5, 2022). Norway is considered to be a high-trust society (e.g., Torcal, 2017), and public trust in the public health authorities remained high throughout the pandemic but with somewhat lower levels during particular phases (Norwegian Directorate of Health, 2022). We, therefore, decided to study the discussion on Twitter during three periods with invasive infection control measures that included partial lockdowns (March–May 2020, November–January 2021, and November–January 2022) (see the methods section for more details). We pose the following research questions:

**Research Question 1:** How did Twitter users perceive the trustworthiness of the Norwegian political leadership and Norwegian health authorities concerning the handling of the COVID-19 pandemic?
**Research Question 2:** Which aspects of trustworthiness were considered important and how did this evolve over time in this context?

Next, we provide a brief overview of the theoretical perspectives we draw on. This is followed by a short section on methods before we present the analysis focusing on the three mentioned periods. A final section draws the findings together and relates them to the broader topic beyond the empirical basis for the article.

## Trust, Trustworthiness, and Social Media Dynamics

Trust is singled out as paramount for public health authorities to secure adherence to health advice during a pandemic like COVID-19 (e.g., Majid et al., 2021; Siegrist & Zingg, 2014). There are, however, many different conceptions of trust. In sociology, for instance, trust has been defined as “a bet about the future contingent actions of others” (Sztompka, 1999, p. 25). In political science, a perspective on moral has also been added, seeing trust as based on the assumption that others share some moral values with us, thus they will not take advantage of us (Uslaner, 2002). Yet another strand of research within political science builds on rational choice theory and sees trust as a form of encapsulated interest—“the potentially trusted person has an interest in maintaining a relationship with the truster, an interest that gives the potentially trusted person an incentive to be trustworthy” (Hardin, 2006, p. 17). In the present study, however, we rely on the definition of trust most frequently found in organizational research (Searle et al., 2018):

*the willingness of a party to be vulnerable to the actions of another party based on the expectation that the other will perform a particular action important to the trustor, irrespective of the ability to monitor or control that other party* (Mayer et al., 1995, p. 712, original italics).

While many studies have focused on the micro level of interpersonal trust, research can also be found at the meso level of institutional and organizational trust, or the macrolevel analyzing levels of trust in a society or trust in, for instance, science (Valentini et al., 2022). At the individual level then, trust is a psychological state of willingness to be vulnerable based on positive acceptations of others. At a higher, social level, trust concerns the *shared* psychological state of the same. In the context of the present study, the general trust issue relates to how the public health authorities and the political leadership are expected to manage the pandemic and provide sound health advice.

Building on the literature on trust from organizational research, it is common to separate between two antecedents of trust—*trusting dispositions* and *trustworthiness* (Baer & Colquitt, 2018; Mayer et al., 1995; Rousseau et al., 1998). A trusting disposition is a stable tendency of the trustor to rely on what is said and done by others (Baer & Colquitt, 2018). A trusting disposition means that you are willing to ascribe good intentions to other people.

Still, while the disposition to trust has some explanation value, people do not trust everyone to the same extent across different situations or related to different tasks or topics (Baer & Colquitt, 2018). Thus, attention is directed toward impressions of the trustworthiness of the trustee. As mentioned in the introduction, trustworthiness can be defined as a construct with three elements (Mayer et al., 1995):

- Ability, understood as the knowledge, skills, expertise, and competencies required to perform in a specific domain or to tackle some specific tasks.
- Benevolence, defined as the extent to which a person believes the other is concerned about his or her well-being, without recurse to self-interested motives. Responsiveness, caring, and loyalty are keywords frequently brought up.
- Integrity, here taken to mean that the trustee adheres to a set of values shared or accepted by the trustor. This is often operationalized as the belief that there will be consistency between word and deed.

Within organizational research it is concluded that these three facets are strongly correlated to trust, and indeed that trust is “primarily and essentially a function of perceived trustworthiness” (Baer & Colquitt, 2018, p. 170). Interestingly, the ancient rhetorical tradition, with Aristotle (2007) as a key figure, suggested very similar ways to strengthen *ethos—*the revelation, construction, or projection of character through speech (Baumlin & Scisco, 2018). Ethos is enhanced by the demonstration of practical wisdom, goodwill, and virtue (Aristotle, trans. 1991). “Practical wisdom” has been taken to mean that the rhetoric should inhabit good sense, expertise, and come across as intelligent and knowledgeable. Similarly, “goodwill” is whishing good for others for their sake and is communicated by, for instance, holding some of the basic aspirations of the audience, speaking their language. Finally, “virtue” relates to the moral character of the speaker (Kinneavy & Warshauer, 1994). Given the chronological, political, and cultural distance from ancient Greece, it is rather surprising that modern empirical research on *credibility* has arrived at components that “remain resiliently Aristotelian” (Baumlin & Scisco, 2018, p. 206). While not wanting to conflate or underplay the differences between these theoretical streams, we do perceive some striking similarities. Still, in the remaining part of the study, we will primarily draw on the tradition of organizational research.

As mentioned, one study of the COVID-19 pandemic singled out *ability* as crucial for the trustworthiness of experts (van Dijck & Alinejad, 2020, p. 2). Other studies of the pandemic and trustworthiness have focused on traits that have closer links with *integrity*: Politicians as a group are often characterized as untrustworthy, as they have political agendas and strategies that are not founded on science (Hendriks et al., 2022; Janssen et al., 2021). Public health authorities, on the other hand, were perceived as more trustworthy, presumably lacking a political agenda. Utilizing the above model, yet another study pointed to how factors such as personal contact with experts, as well as the perceived independence from political elites, might increase perceptions of trustworthiness (Mihelj et al., 2022).

Again, context needs to play an important role when studying trustworthiness and thereby trust (Mayer et al., 1995). For example, seeing that ability is domain-specific, the perceived ability may change as the dynamics of the situation relevant to the task at hand change. Furthermore, a decision may appear inconsistent with earlier decisions and thereby lead to questions about integrity. However, knowledge about the context of the decision—that the trustee had no other option—can lead to perceptions of high integrity. Thus, the changing situational aspects necessitate an analytical focus that includes time, in line with the call for research that improves our understanding of situational dynamics of trustworthiness (Baer & Colquitt, 2018).

During the COVID-19 pandemic, the relevance of social media like Twitter has been demonstrated as researchers, politicians, and citizens alike share information and discuss the different aspects of the pandemic (Chen et al., 2020; Rao et al., 2021). One study shows how influential Twitter users can reduce uncertainty by providing accurate information as a preventive action against rumors, misinformation, and disinformation during a crisis (Mirbabaie et al., 2020). Looking at social media affordances, one study finds that Twitter has a negative effect on conspiracy beliefs (Theocharis et al., 2021). The exchanges between users are public and often between strangers, which in turn counteracts echo chambers and exposes conspiracy theories to public scrutiny.

As a platform, Twitter can strengthen the position of authorities as experts but also promote alternative forms of expertise. Kjeldsen et al. (2022) studied negotiations of trust and expert trustworthiness in connection with a Norwegian debate program at the outset of the COVID-19 pandemic. Analyzing tweets about the program, they found a great deal of trust in the health authorities. The Twitter activity as a whole reinforced the authorities’ expert status but also included several other positions supporting alternative expertise. In Finland, Väliverronen et al. (2020) identified a polarization with increased contestation of the health expertise of the authorities during the COVID-19 crisis. The authors argue that a new form of alternative networked expertise on Twitter emerged as a result (Väliverronen et al., 2020). COVID-19-related debates on Twitter thus contain a wide span of opinions, ranging from support of the authorities to contestation of the authorities’ expertise and promotion of alternative expertise and alternative political views. Other studies (e.g., Vicari, 2017) have also pointed to the positive emergence of non-elite actors on Twitter and to the need to understand the related discursive practices. In this context, how trustworthiness is negotiated by experts, politicians, and lay people alike in the Twitter-sphere.

## Data and Method

The data material for the study was retrieved through the Twitter API for Academic Research. For scraping relevant data, we established a keyword list in Norwegian with words related to COVID-19, government, politicians, health authorities, and infection control measures. Tweets including one or more of the keywords were then collected and parsed. For the analysis of the data, we used a tool developed at the home institution of two of the authors (Grøtan et al., 2020). The tool allows researchers to search for relevant posts by filtering the data set for time and search words. Moreover, the tool has a user interface that displays tweets with their content, replies, mentions, retweets, number of likes and shares. This enables content analysis of a tweet in its Twitter context.

Although only 28% of the Norwegian population have a Twitter profile, and 10% use Twitter daily (Ipsos, 2023), we have chosen to focus on Twitter as it is an important part of the networked sphere in which political issues are discussed (Breslin et al., 2020). Twitter is often used by politicians, journalists, and citizens generally more interested in politics than the average person (Robinson, 2022). Furthermore, social media data provide the opportunity to analyze people’s authentic and unfiltered responses to a given issue (Orth et al., 2020).

Considering that perceptions of trustworthiness are situational contingent and that different phases of the pandemic require different responses (Mølster & Kjeldsen, 2021), we have chosen to study three periods with Twitter data, focusing on the most invasive infection control measures in Norway during the COVID-19 pandemic. The first partial lockdown was from 12 March 2020 to 12 May 2020, the second period was from 5 November 2020 to 31 January 2021, and the third period was from 15 November 2021 to 15 January 2022. The periods are described further in the analysis section.

From a pool of nearly 2.3 million tweets,^1^ we filtered data using groups of search words in Norwegian thematically focused on restrictions and infection control measures as well as for Norwegian health authorities.^2^ We distinguished between the political leadership (the Ministry of Health and Care Services, the Minister of Health, and leading politicians in the government such as the Prime Minister) and the health authorities (the Norwegian Institute of Public Health [NIPH] and the Norwegian Directorate of Health) with related public representatives.

The results from the filtering included posts and comments that contained words related to infection control measures and authorities. In addition, we filtered the data for date ranges corresponding to the three periods in question. From a corpus of 6046 tweets, we excluded tweets that concerned other policy areas, duplicates, tweets posted by the authorities themselves and news media, as well as tweets tagging the public health authorities or political leadership but mainly addressing issues other than the pandemic. In total, we ended up analyzing 2824 tweets, including comments. The distribution is shown in Table 1:

**Table 1.** Sum of Value by Period and Authority.

	Political leadership	Health authorities	Grand Total
Mar 20–May 20	222	619	841
Nov 20–Jan 21	171	267	438
Nov 21–Jan 22	687	858	1545
Grand total	1080	1744	2824

To investigate the research questions, we performed a quantitative content analysis (Krippendorff, 2018). Considering the importance of grasping temporal dimensions, the content analysis enabled a better understanding of the evolvement of the different aspects Tweeters considered important. For this purpose, we established a code book focused on the three trustworthiness aspects of ability, integrity, and benevolence, with consideration of sentiment (positive/negative). In this regard, some studies highlight that one dimension is more important in certain situations than others (e.g., Colquitt et al., 2011). While it is difficult to code these aspects of trustworthiness, we based our scheme on previous studies that categorize trustworthiness (Baer & Colquitt, 2018; Mayer et al., 1995). In addition, following initial sample coding, the code book was extended and adapted to capture nuances and provide context for the analysis. This coding was performed by two of the researchers and a research assistant. If an alternative value had been assigned to one of the variables, a joint review was performed to decide which category was the most suitable. Ultimately, we added two categories, namely, neutral/informative and critical/investigative.

*Ability* covers tweets with expressions of trust (positive sentiment) and distrust (negative sentiment) of the authority’s expertise and competence. *Integrity* covers expressions of trust (positive sentiment) and distrust (negative sentiment) in the authority being transparent, honest, or fair. While *benevolence* covers tweets concerning the motives of authorities—that they are perceived to want the best for the public (positive sentiment), or that authorities are taking actions for own gain (negative sentiment).

*Neutral/informative* tweets inform others about decisions and regulations as well as neutrally formulated questions. This category reflects the functioning of Twitter as a platform where people are looking for news and often take on the role of informing others (Theocharis et al., 2021).

*Critical/investigative* tweets are about decisions and regulations with a more critical tone as well as critically formulated questions or suggestions (lay expertise) aimed at the authorities. These tweets are not demonstrating distrust but are posing questions about policies and decisions made. Following a strict delineation between the categories, the difference between critical/investigative and negative ability, for example, was determined based on how the tweet was formulated and its sentiment. The argument for such an approach is that a person who tweets questions about authorities’ actions does not necessarily have a negative perception, but seeks to offer different perspectives and to encourage debate.

Frequent team meetings were held to discuss the code book and how to code in the most consistent manner. The data material was then coded by one of the researchers examining each entity (political leadership, the NIPH and the Directorate of Health) and period. Because tweets are limited in length and may be written in a sarcastic or humoristic tone—which is often difficult to discern—we operated with a strict understanding of the categories for the coding. Only tweets where the ability, integrity, or benevolence of the authorities (represented by an official or the authority/entity itself) was clearly targeted and described with a clear positive or negative sentiment were coded in the related trustworthiness categories. Following our strict interpretation, we have operated with mutually exclusive categories, and no tweets were coded with two or more categories. The coded data material formed the basis for an evaluation of situational aspects and topics addressed, allowing for sensitivity of context.

To assess coding agreement, intercoder reliability was calculated by double-coding a random subsample of the data (*n* = 284 or 10 %). We used Cohen’s Kappa that factors out agreement due to chance and is used for nominal and mutual exclusive categories. We obtained an average reliability of .63, indicating substantial agreement (Sim & Wright, 2005).

## Analysis

**Figure 1.**
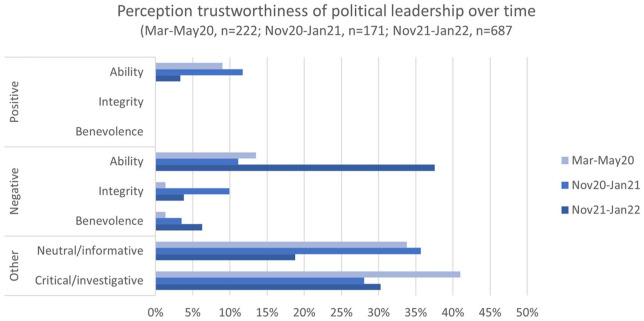
Positive and negative posts related to the trustworthiness of political leadership comparing three periods.

**Figure 2.**
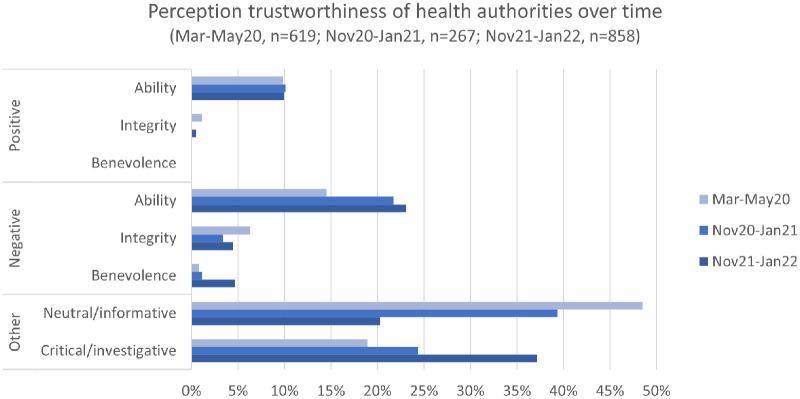
Positive and negative posts related to the trustworthiness of health authorities comparing three periods.

Overall, the analysis shows that trustworthiness is not static but something that evolves over time. Moreover, we observe that ability is the most discussed trustworthiness aspect in our material. When issues related to integrity and benevolence are commented, it is mainly in negative terms. Interestingly, in our case, these observations of how trustworthiness is discussed are similar for both the political leadership and the health authorities as can be seen in Figures 1 and 2 that show the distribution for the political leadership and health authorities, respectively.

In the following, we address each period more in-depth. The aim is to analyze how Twitter users perceived the trustworthiness of the political leadership and health authorities and to study the processes as they relate to specific situations with their own dynamics and how they unfold over time.

### Period 1: Lockdown Reactions

On 12 March 2020, the Norwegian government announced a partial lockdown of the country due to the COVID-19 virus. These were the strongest restrictions imposed on Norwegian society since World War II. The government temporarily closed schools and universities, as well as borders to neighboring countries, and large social gatherings were forbidden.

#### Political Leadership

In this first period, Twitter users are neither overly positive nor negative when commenting on the handling of the pandemic. They are mainly commenting on and discussing different measures and which actions should be taken. The most frequently mentioned trustworthiness concern relates to ability—the first of the three facets of trustworthiness (Mayer et al., 1995). These Tweets express that the users do not trust the government, that the measures are irrelevant or terrible, and that restrictions should not be national but rather target areas with high infection rates. Such criticism and lack of trust in politicians have also been found in other studies (Hendriks et al., 2022; Janssen et al., 2021). Still, as shown in Figure 1, there are also positive tweets related to ability. These typically express praise of a job well done and that the Twitter users feel safe in the hands of the government.

There are also a few tweets related to the two other trustworthiness aspects—integrity and benevolence (Mayer et al., 1995). Examples of the former include how the Minister of Health is accused of not speaking the truth, and it is claimed that government employees are exempted from restrictions. An example of the latter includes allegations that actions are taken for political gain, and not in the interest of citizens. All the tweets related to integrity and benevolence are negative.

Nevertheless, the largest category is critical/investigative tweets. Without being negative toward authorities, Twitter users argue, for instance, that the border to Sweden should be closed and that face masks should be mandatory. The Twitter platform is used to present own opinions of how the situation could be handled. This then points to the peculiar trait of networked “expertise” ushered in by social media and also found in other studies (Väliverronen et al., 2020). Moreover, there are also many neutral/informative tweets in which Tweeters inform others about new measures, press conferences, and convey news from government officials.

#### Health Authorities

Figure 2 shows that most of the Tweets mentioning the health authorities in Period 1 are neutral/informative, in contrast to Tweets about the political leadership. These tweets contain neutrally formulated questions as well as communication about news and information from health authorities. The critical/investigative tweets indicate that people are concerned and confused about restrictions, such as quarantine rules, and that they question the usefulness of various restrictions. Thus, most tweets related to health authorities in this period are about informing others.

The most frequently mentioned trustworthiness factor is related to ability, thus echoing at least one previous study of COVID-19 (i.e., van Dijck & Alinejad, 2020). In our material, people tend to be negative toward the NIPH’s provisions on quarantine and face masks, and argue that the measures are too lax. The Directorate of Health is judged as incompetent after the leadership was infected and thereafter exempted from quarantine rules. The reasoning behind measures is questioned, with a perception of weakened credibility. But there is also support for health authorities’ ability and professional authority, both implicitly (by referring to their statements or information from the site or saying: “According to the NIPH. . .”), as well as explicitly (e.g., pointing out that the work by the Assistant Director of Health is impressive).

Posts related to integrity and benevolence are predominantly negative. Tweets concerning integrity criticize the fact that face masks were not recommended by the health authorities. This is associated with a lack of equipment instead of being the ideal professional assessment. Twitter users also insinuate that the health authorities may be under political pressure. However, there are also a few positive comments concerning integrity, which praise health authority experts for being open about uncertainty and dilemmas. Those who are negative in terms of benevolence ask which interests the health authorities are really protecting, suggesting hidden motives that are not in the interest of the public. However, overall, there are few instances of tweets promoting conspiracy theories. This has also been reflected in other studies demonstrating high levels of trust in both the public health authorities and the political leadership in Norway during COVID-19 (e.g., Wollebæk et al., 2022).

### Period 2: Continued Restrictions and Vaccination

After a summer and autumn with low infection rates and few restrictions, the infection rate started to rise at the end of autumn 2020. The Norwegian government and the health authorities started reapplying strict infection control measures by the end of October (Office of the Prime Minister, 2020). Additional measures were introduced the following month, including restrictions on the number of household guests and the number of people at public events.

This second wave of infections coincided with an increased infection rate in Europe and the pending approval of various COVID-19 vaccines (Norwegian Government, 2021). Despite the restrictions applied, the infection rate continued to rise from November to January 2021. At the same time, the first vaccine shot was administered on 27 December 2020 (Norwegian Ministry of Health, 2020).

#### Political Leadership

While we are entering a second period of strict measures, there are few major differences in Twitter patterns compared to the first period. Regarding trustworthiness, the largest category continues to be about ability though with a slight rise in positive tweets. Tweeters express satisfaction with the infection control measures and appreciation of the government. Moreover, the political leadership is praised as a great leadership that has succeeded in saving many lives. Negative tweets concerning ability, however, consider the political leadership as not fit for the job. The inconsistency in the regulations, with strict quarantine rules for travelers that enter Norway, is contrasted with the lack of testing and follow-up of the foreign workers entering the country to work. Political leaders are also chastised for the slow vaccination pace and for lacking a clear vaccination strategy. Some Twitter users argue that the COVID-19 regulations are too lenient, while others consider them too strict.

While there is an increase in positive ability perceptions, there is also a clear rise in negative tweets that fall under the category of integrity. This might be explained by what Norwegian Tweeters call the general lack of honesty of the Minister of Health. The argument is that his use of the positively loaded word “dugnad” (meaning making a voluntary common effort) is a way of masking the reality of living with strict regulations. This again might suggest a form of fatigue, although the overall change from Period 1 is not substantial. Another accusation is that he ignores recommendations from the health authorities due to pressure from business interests. The criticism has a conspiratorial tone when the benevolence dimension is involved. The political leaders are accused of being globalists who are using restrictions and vaccines to implement dictatorship. Still, it is important to emphasize that such tweets are few and far between.

Interestingly, in Period 2 most of the tweets are neutral/informative and mainly contain retweets of information from the Twitter account of the Ministry of Health, information about restrictions and regulations in other countries, neutrally formulated questions, and factual discussions between Twitter users. Within the critical/investigative category, we find questions about infection control measures and specific suggestions for new restrictions. Others question the efficiency of the newly imposed rule of a compulsory stay at a quarantine hotel for travelers entering Norway from abroad.

#### Health Authorities

Compared to the first period, there is a notable rise in negative tweets concerning the ability of the health authorities and a corresponding decline in neutral/informative tweets. This again points to the importance of researching the dynamic, situational aspects of trustworthiness (Baer & Colquitt, 2018). Simultaneously, the largest share of posts is still neutral/informative, and the debate is marked by factual discussions. Concerning the perception of trustworthiness, the positive tweets about the ability consider the health authorities to be a trustworthy source of information and express support for the NIPH’s face mask recommendations and defend them against criticism. However, the most common articulations criticize the health authorities for their recommendations being too lenient, for moving too slow before deciding on new infection control measures, for non-scientific based recommendations, and for a slow vaccination pace.

All tweets under integrity and benevolence are negative in this period. Concerning integrity, two topics are especially prevalent and create debate around the sincerity of the health authorities. These are the earlier recommendation of the NIPH not to use face masks, and high-profile employees’ comments on herd immunity on Twitter, topics that the NIPH changed their standpoint on during the first year of the pandemic. This change is judged as a possible lack of integrity, and Twitter users call for transparency on the matter. Further, the Directorate of Health’s appearance in a debate program where their representative was unable to provide clear answers led Tweeters to question the basis for their recommendations. Concerning negative tweets that fall under the category of benevolence, the health authorities are accused of using the infection control measures to remove democracy, and for enjoying the pandemic as their moment to shine.

Neutral/informative tweets mainly contain information about infection control measures in Norway and abroad, as well as neutral questions. Critical/investigative tweets, on the other hand, mainly question the direction of measures. Some Tweeters argue that there is no time to wait for health authority researchers before making decisions, while others demand more information about infection numbers.

### Period 3: After the Re-Opening Party

On 24 September 2021, the government announced a reopening of Norwegian society. The decision was based on sinking infection rates and a flattening of the curve of hospitalizations (Office of the Prime Minister, 2021). The decision was met with high public enthusiasm. Shortly before, the September election introduced a new left-center government that soon faced rising infection rates and the new Omicron variant of the COVID-19 virus. The government introduced travel restrictions, followed by recommendations of social distancing. In December, more restrictive measures were in place such as ban on serving alcohol and mandatory face mask use (Norwegian Ministry of Health, 2022).

#### Political Leadership

By the third period of lockdown, we observe a notable negative trend for the political leadership in terms of trustworthiness. Indeed, the largest category in this period overall is ability. These tweets are predominantly negative. A recurring notion is that the government is too passive and that government officials need to understand the seriousness of the situation. Notably, many argue that the previous government did a better job in handling the pandemic. Hence, the content of these tweets suggests that the shift in government played a role in this change as well as there is a sense of fatigue from the crisis. Those who tweet positively about their ability mainly appreciate that the government is avoiding implementing restrictions based on panic.

When it comes to tweets concerning integrity and benevolence, all are negative. Tweets on integrity center around alleged abuse of power and that the political leadership is lying. Often, the government is criticized for withholding information and not being open about the basis for its decisions. Those with concerns about benevolence tweet that the government is not doing what is best for the citizens and that politicians are more concerned with saving money. Interestingly, there is a shift in this category during the third period, where opinions range widely from people being critical that the government has not introduced restrictions (which impacts citizens negatively) to others being critical when the government is implementing restrictions (arguing that a closed society only benefits the wealthiest).

Many Twitter users question the political leadership’s handling of the situation without commenting on trustworthiness. Like the abovementioned categories, during the third partial lockdown, there is a divide among the critical/investigative tweets, between those who question the need for restrictions when most are vaccinated, and those questioning the lack of measures and restrictions.

#### Public Health Authorities

Interestingly, there is a similar trend regarding public health authorities as for the political leadership. There are more negative posts related to trustworthiness compared to the previous periods, as well as more Twitter users that are critical. Another similarity is the mixture of those who want stricter measures and those who seek a restriction-free society. Thus, it appears that as time passes, the different stances toward restrictions become more evident among Tweeters.

The most frequently mentioned trustworthiness issues are related to ability and are predominantly negative. Negative tweets criticize recommendations for being illogical and state that the argumentation is based on insufficient grounds and that the health authorities have undeserved trust. Positive tweets on ability entail both direct and indirect support of the competence and expertise of the health authorities. Some tweet that the health authorities are sensible and that their recommendations are reasonable, while others are more indirect, criticizing the government for not listening to the experts.

In terms of trustworthiness, issues related to integrity and benevolence are, again, predominantly negative. On integrity, Tweeters express concerns that health authority experts are withholding information or lying and that it is the professional expertise that controls the country—not the political leadership. Those tweeting in terms of benevolence state that the measures and restrictions are disproportional, harmful to kids, and destructive for society. Moreover, the health authorities are accused of earning money on the pandemic and therefore seek to prolong it. Overall, the content of the negative tweets regarding trustworthiness underlines a sense of fatigue.

As can be seen in Figure 2, the largest category in this period is tweets categorized as critical/investigative. There is a notable increase compared to the previous periods, and a corresponding drop in the category of neutral/informative tweets. Within the critical/investigative category, central issues are the proportionality of measures, questions about the NIPH’s turnarounds on advice for face masks—as well as criticism of different messages being conveyed by the Directorate of Health and the NIPH, which is said to be confusing.

## Discussion and Conclusion

The main contribution of this study lies in the demonstration of how one particular situational dynamic, time, plays a role in negotiations of trustworthiness on the social media platform of Twitter in a pandemic like COVID-19. This answers a call in the literature relating to trustworthiness in general (Baer & Colquitt, 2018) as well as the call for more studies of the temporal aspect of social media discussions (Vicari, 2017). Our main findings can be summed up as follows:

First, we showed how both the political leadership and the health authorities received more negative tweets than positive ones regarding trustworthiness. The difference is most notable in the third period, but not substantial in the two first periods. Thus, the Twitter users in this sense did not appear to be in line with the largely positive attitudes demonstrated in the weekly surveys that the Norwegian Directorate of Health (2022) ran during COVID-19. Still, it must be remarked that there were also positive expressions of support in our data set, thus illustrating that social media like Twitter is not necessarily all about negativity (Ceron, 2015; Humprecht et al., 2020).

Second, using the trustworthiness framework focusing on ability, integrity, and benevolence (Mayer et al., 1995), we showed how the largest group of Twitter users commented on *ability*, both those with positive and those with negative perceptions. This pattern is similar for the political leadership and health authorities alike. Ability also figures as a prominent factor in other studies (van Dijck & Alinejad, 2020). The political leadership was judged on the relevance and consistency of measures, given the situation, its ability to handle the pandemic, and its understanding of the situation. The health authorities were evaluated on the content and timing of recommended measures and their professional authority and credibility.

Users commenting on integrity and benevolence overwhelmingly cited negative concerns. Regarding integrity, central issues were whether the methods and basis for political decisions were transparent. While tweets citing concerns about benevolence suspected hidden motives, and that the handling of the pandemic was not designed to benefit citizens. Notably, most tweets with conspiratorial content were found in this category, linking to a discourse analyzed elsewhere (e.g., Erokhin et al., 2022). Overall, however, there were few tweets with conspiratorial content, strengthening the finding that Twitter affordances might counteract the spread of conspiracy theories (Theocharis et al., 2021).

Third, while there are similar trends across the three periods of partial lockdowns, there is a notable development in Period 3. Compared to the two first periods, there is a prominent increase in negative concerns over ability with regard to the political leadership. Analyzing the cited concerns indicate that the change in government had a negative impact on the perception of trustworthiness, as well as a sense of fatigue, something that has been demonstrated by other scholars as well (e.g., Su et al., 2022). The quantitative content analysis also shows how perceptions of trustworthiness on Twitter are formed by context and events. For example, there is a notable shift between those who are criticizing measures for being too lenient and those arguing that measures are too strict, depending on the situation at hand and the actions by the political leadership and health authorities. There is no clear consensus on this matter, with Twitter users leaning toward each of these opposite perceptions during all three periods, although the divide is most pronounced during the third period. This change strengthens the argument that trustworthiness must be analyzed over time (Baer & Colquitt, 2018), that different phases condition different responses, and that each situation is unique with its own dynamics (Mølster & Kjeldsen, 2021).

Fourth, surprisingly, there are few differences in perceptions of trustworthiness when comparing the political leadership with the health authorities. While previous research has found health authority experts to be perceived as more trustworthy than political leadership (Hendriks et al., 2022; Janssen et al., 2021), there are mostly similarities in our study. Instead, the differences pertain to how more users tweet neutral questions or informative posts to health authorities, whereas there are more critical/investigative tweets in relation to the political leadership. Politicians as a group are thus subject to more scrutiny overall, albeit the differences in perceptions of trustworthiness are marginal. As underlined by Mihelj et al. (2022), the perceived independence from political elites can increase perceptions of trustworthiness. In our case, we observed accusations of close ties between the two spheres, with some insinuating that the health authorities are governing the country and seeking to prolong the pandemic to maintain their influence. Thus, the lack of perceived independence can help explain the decline in the perceived trustworthiness of the health authorities as the pandemic unfolded.

Fifth, the analysis shows how trust, distrust, praise, and criticism take different forms. Moving beyond the issue of trustworthiness, we found that a large part of the analyzed tweets can be categorized as either critical/investigative or neutral/informative. This suggests that rather than expressing trust or distrust, these users utilize Twitter as a platform to raise their own ideas for an improved handling of the pandemic, or to inform others on various issues and perspectives. The informative part of Twitter can be considered to contribute to reducing uncertainty (Mirbabaie et al., 2020) but also to boosting the trustworthiness of the authorities by retweeting their expertise (Kjeldsen et al., 2022). Moreover, while those citing critical/investigative concerns do not criticize the authorities directly, they often point out that the authorities have missed something, and that other considerations should have been taken into account. While not directly criticizing ability, the ability of the health authorities is challenged. Thus, many Twitter users assume an “I know better” position, promoting their lay expertise. In general, the size of these two categories demonstrates the affordances of Twitter—that the platform is mostly used by those looking for news, and that the networks are open and asymmetrical (Theocharis et al., 2021). What we observe is a broad group of Tweeters that aim to contribute to the debate with other perspectives, rather than being necessarily negative toward the public authorities and political leadership. This also ties in with findings from studies emphasizing how alternative networked expertise might emerge on Twitter (Väliverronen et al., 2020).

Taken together, this article sheds light on how the dynamics of social media are important when studying trustworthiness in a protracted crisis. The study suggests that Twitter is an arena for negotiations over ability in particular and that discussions of ability can intensify as a crisis drags on. For public health authorities, the insights on how discussions on trustworthiness unfolded over time on Twitter can aid in developing more detailed and context-sensitive communication strategies (van Dijck & Alinejad, 2020, p. 8). The results highlight how ability is the most discussed aspect of trustworthiness, which is definitely an area where the public health authorities would be expected to have the upper hand if only based on their expertise network and experience of handling previous pandemics (Kjeldsen et al., 2022). The fact that so much lay expertise was offered by Twitter users leads to the tempting conclusion that the public health authorities should be relying more on Twitter and put special emphasis on invitational expertise rhetoric engaging in dialogue (Kjeldsen et al., 2022). Also, the ability to share tweets would provide a possibility for so-called third-party strategies to strengthen trustworthiness and cultivate relationships with supportive Tweeters. While research has pointed to how content on social media such as Facebook and YouTube can strengthen conspiracy theories, the “asymmetrical structure of connections” on Twitter “has a negative effect on conspiracy beliefs” (Theocharis et al., 2021, p. 18).

This article was based on empirical material from Twitter, which means that the study has certain limitations. Because we lack demographic data, we cannot generalize the findings to the population. Moreover, as noted, few Norwegians use Twitter daily. At the same time, Twitter is used by opinion leaders and people generally more interested in politics than the average person. It would be interesting to add a network analysis to explore how aspects of trustworthiness are negotiated in certain clusters focusing on certain aspects. In the Norwegian setting, discussions of trustworthiness primarily relate to ability. Obviously, the situation might be different in countries characterized by low levels of trust. Further studies could be carried out in such contexts and explore issues beyond COVID-19. Considering the much-discussed change of ownership of Twitter in October 2022, it would also be interesting to see how Twitter dynamics play out in this new context.

## Notes

1. The data collection is approved by Sikt (ref. 691669), in addition to an approved Data Protection Impact Assessment.

2. The search terms were: “cabin prohibition,” “restrictions,” “lockdown,” “infection control measures,” “quarantine hotel,” “quarantine,” “measures,” “close contacts,” “facemasks,” “prohibition,” “one meter,” “ban on serving alcohol,” “FHI,” “Institute of Public Health,” “Stoltenberg,” “camisto,” “prebens,” “epidemino,” “Directorate of Health,” “Guldvog,” “Nakstad,” “fungass,” “director of health,” “Ministry of Health,” “Minister of Health,” “health authorities,” “government,” “prime minister,” “Høie,” “BentHHoyre,” “Solberg,” “Kjerkol,” “ingvildkjerkol,” “Støre.”
